# Optimizing dietary crude protein, branched-chain amino acids, and energy levels for broiler performance using a Box-Behnken design

**DOI:** 10.1016/j.aninu.2025.02.006

**Published:** 2025-04-25

**Authors:** Sosthene Musigwa, Pierre Cozannet, Mingan Choct, Shu-Biao Wu

**Affiliations:** a Animal Science, School of Environmental and Rural Science, University of New England, Armidale, NSW 2351, Australia; b Center of Expertise and Research in Nutrition (CERN), Adisseo France SAS, 92160 Antony, France

**Keywords:** Reduced-crude protein, Fat pad content, Apparent metabolizable energy, Net energy

## Abstract

This study aimed to investigate the limitations and interactive effects of dietary crude protein (CP; 15%, 17% and 19%), excess branched-chain amino acid (BCAA) inclusions (0%, 20% and 40%), and net energy (NE; 9.0, 9.7 and 10.4 MJ/kg) on performance and nutrient utilization for Cobb 500 mix-sex broilers, using a Box-Behnken design. The study consisted of 2 experiments: Exp. 1 involved 1092 chickens, and their performance was measured from d 19 to 35, and Exp. 2 employed 156 birds for NE measurements from d 25 to 28. Both experiments used the same diets (*n* = 13), each replicated 7 times for Exp. 1 and 6 times for Exp. 2. On d 35, 4 birds per pen (2 males and 2 females) were sampled to collect ileal digesta and weigh carcass parts. Feed intake (FI), NE intake (NEi), weight gain (WG), feed conversion ratio (FCR) and breast yield were affected by CP × NE (*P* < 0.001) and CP × BCAA (*P* = 0.041). Diluting NE in the reduced CP (RP)-diets led to a higher FI (*P* < 0.001) and breast yield (*P* < 0.001) than high NE, but the NE levels had no effect (*P* > 0.05) on FI and breast yield in high CP (HCP)-diets. Breast yield increased (*P* = 0.041) with BCAA in HCP-diets, whereas in the RP-diets, the yield lowered (*P* = 0.041) with increased BCAA. A similar trend was observed with fat content: in HCP-diets, fat content increased (*P* = 0.007) with BCAA, while in RP-diets, fat content decreased (*P* = 0.007) with higher BCAA levels. In addition, increasing BCAA in the RP-diets decreased (*P* < 0.001) FI, NEi, WG and increased FCR (*P* = 0.001) than low BCAA. However, BCAA levels had no effect (*P* > 0.05) on these measurements in HCP-diets. High NE increased (*P* < 0.001) NEi and decreased (*P* < 0.001) FCR compared to low NE in the HCP-diets. However, the NE effect on both measurements did not differ (*P* > 0.05) in RP-diets. These results indicate that increasing BCAA levels beyond the recommended amounts in RP-diets impairs energy utilization, leading to poor performance.

## 1 Introduction

The practice of reducing dietary crude protein (CP) content has received considerable attention in the poultry industry as a way of sustainable broiler production. However, substantially reducing CP concentration beyond a certain level in feeds is often associated with compromised growth performance and increased body fat accretion. A high dietary energy intake is often considered to increase lipid accretion, especially in reduced protein (RP)-diets (Chrystal et al., 2020; Parr and Summers, 1991). It was demonstrated that increased body fat accumulation is associated with a higher energy-to-digestible protein ratio in the RP-diets (Collin et al., 2003). Nevertheless, Kamran et al. (2008) found that while maintaining a constant energy-to-digestible protein ratio in RP-diets addressed the fat accretion issue, it did not alleviate the depression in growth performance.

A body of literature emphasized that supplementing RP-diets with branched-chain amino acids (BCAA), i.e., leucine (Leu), isoleucine (Ile), and valine (Val), above the required recommendations can improve broiler performance and reduce fat accretion (Greenhalgh et al., 2022; Maynard et al., 2021; Selle et al., 2020; Zeitz et al., 2019b). In fact, BCAA account for one-third of total muscle protein (Kim et al., 2022). They also make up nearly half of the total supply of dietary essential amino acids (EAA) (Chrystal et al., 2018). BCAA regulate protein synthesis and turnover and both protein and lipid metabolism in broilers (Chrystal et al., 2022; Maynard et al., 2021). Among BCAA, Leu stimulates protein synthesis via the mammalian target of the rapamycin (mTOR) signaling pathway when the availability of all other amino acids (AA) is not limiting (Deng et al., 2014; Zeitz et al., 2019b). However, research has shown that excessive Leu in diets deficient in Ile and Val can adversely affect broiler performance (Kidd et al., 2021a; Waldroup et al., 2002). While concerns about BCAA antagonisms are unlikely in broilers fed practical diets where AA are supplied above the minimum requirements, the risk increases in RP-diets with inadequate individual BCAA ratios. In such diets, excess Leu could induce the catabolism of Ile and Val, especially when these two AA are marginal or limiting (Kim et al., 2022; Maynard et al., 2021; Selle et al., 2020; Waldroup et al., 2002).

Wu (2014) proposed a Leu-to-Lys ratio of 109 for all bird ages, which is lower than the Leu requirements for the growing pig despite this AA contributing equally to whole-body protein in both species. This has raised concerns about the potential underestimation of Leu needs in poultry (Chrystal et al., 2018). Additionally, increasing Leu and Val levels have been suggested to mitigate abdominal fat accumulation and enhance feed conversion ratio (FCR) in broilers offered RP-diets (Chrystal et al., 2018; Yin et al., 2020). Therefore, the current study investigated how dietary CP, BCAA and net energy (NE) levels interact and influence broiler growth responses, fat accretion, and nutrient utilization. It was hypothesized that supplementing BCAA above the requirements would be beneficial to birds fed RP-diets.

## 2 Materials and methods

### 2.1 Animal ethics statement

The experimental procedures for Exp.1 and 2 fully complied with the specific guidelines approved by the animal ethics committee of the University of New England (AEC21-039).

### 2.2 Experimental design

A 3-way Box-Behnken design (BBD) was conducted to assess the effects of CP (15%, 17%, 19%), NE (9.0, 9.7, 10.4 MJ/kg), and excess of dietary BCAA above requirements (0%, 20%, 40%) on growth performance, carcass measurements and nutrient utilization of broilers. The treatment diets were applied from d 19 to 35 post–hatch. The independent variables (factors: X_1_, X_2_, X_3_) and their levels (-1, 0, 1) used in the BBD response surface methodology (RSM) are shown in Table 1, and the resulting 13 treatments are presented in Table 2. The values of the center points for each factor (treatment 7) were 17% CP, 9.7 MJ/kg NE, and 20% excess BCAA. All measurements evaluated vary independently. The study was undertaken in two experiments. Exp. 1 was done in floor pens to evaluate growth performance, carcass traits and nutrient utilization. Exp. 2 was undertaken in closed respiration chambers for NE measurement.

**Table 1:** Ranges of independent variables and their levels.

Item	Levels		
	−1 (low)	0 (medium)	1 (high)
X_1_: CP, %	15	17	19
X_2_: NE, MJ/kg	9.0	9.7	10.4
X_3_: BCAA, % excess^1^	0	20	40

CP = crude protein; NE = net energy; BCAA = branched-chain amino-acids.

1 X_3_ based on ratios of BCAA, to ileal digestible Lys, which was set to 0.995%. These ratios for digestible Leu, Ile, and Val to digestible Lys include, respectively 109, 69, and 80 for 0% excess BCAA; 131, 83, and 96 for 20% excess BCAA; and 153, 97, and 112 for 40% excess BCAA.

**Table 2:** Box-Behnken design for 3 factors each with 3 levels with a total of 13 treatments.

Treatment	Factors (actual level)		
	CP, %	NE, MJ/kg	Excess BCAA, %
1	15	9.0	20
2	15	9.7	0
3	15	9.7	40
4	15	10.4	20
5	17	9.0	0
6	17	9.0	40
7 (center point)	17	9.7	20
8	17	10.4	0
9	17	10.4	40
10	19	9.0	20
11	19	9.7	0
12	19	9.7	40
13	19	10.4	20

CP = crude protein; NE = net energy; BCAA = branched-chain amino-acids.

### 2.3 Diet preparation

The common starter and common grower diets were formulated based on wheat and soybean meal, with 12.45 MJ/kg apparent metabolizable energy (AME) collected of nitrogen (N) or AMEn, 9.87 MJ/kg NE, and 22.00% CP for starter, and 12.66 MJ/kg AMEn, 10.05 MJ/kg NE, and 20.53% CP for grower (calculated, data not provided). The composition and the calculated nutrients of the experimental wheat/barley-soybean meal-based treatment diets are presented in Table 3, Table 4, respectively. The main dietary nutrients from the ingredients were calculated using near-infrared spectroscopy (NIRS). All the diets were formulated to meet or exceed the nutrient specifications for Cobb500 (2018) with the exception of the finisher diets where the levels of CP were 15%, 17% and 19%, NE were 9.0, 9.7 and 10.4 MJ/kg, and BCAA were 0%, 20% and 40% above the recommended requirements. The Lys value of 0.995% utilized in this study was computed as an average of Cobb finisher 1 and finisher 2 (Cobb500, 2018). Subsequently, the remaining AA were calculated based on the Lys ratio to each AA from Texas A&M (Wu, 2014). To avoid BCAA imbalance effects, the Leu:Ile and Leu:Val ratios were fixed to 1.58 and 1.36, respectively, in all treatments by supplementing crystalline Leu, Val and Ile. However, the BCAA-to-Lys ratios in T11 could not be controlled due to the nature of the ingredients used, where all BCAA were bound AA and no crystalline BCAA supplementation was supplemented. Diets with the basal BCAA content (0% excess) were formulated to contain the optimal ratios of Lys to Leu, Val and Ile of 109, 80 and 69, respectively, proposed by Wu (2014). The 20% BCAA contained 131, 83, and 96, and the 40% BCAA had 157, 99, and 115, respectively. The corresponding ideal Lys, Leu, Ile and Val was 0.995%, 1.085%, 0.720% and 0.796% in the 0% BCAA, 0.995%, 1.3015%, 0.824% and 0.944% for the 20% BCAA, and 0.995%, 1.518%, 0.961% and 1.114% for the 40% BCAA. The analyzed AA concentrations in the main ingredients are presented in Table 5, and the analyzed concentrations of CP, AA and energy in experimental diets are shown in Table 6. During feed formulation, all EAA and non-essential AA (NEAA) were accounted for in the feed formulation model, so that a balance between all AA can be determined. To balance EAA and NEAA content, true protein (TP = EAA + NEAA) concept was used and it was calculated using the method described by Alhotan and Pesti (2016) as follows:

Feed protein content (%) = ingredient protein (%) × amount of ingredient used (%),

Ingredient total N (%) = protein content/6.25,

Ingredient TP contribution (%) to feed = ingredient total N × K_A_,

where N = nitrogen; TP = true protein or EAA + NEAA; K_A_ = ingredient specific N to protein conversion factor sourced from the literature (Krul, 2019; Mosse, 1990). Where applicable, NEAA were added to adjust the ratio of EAA to true protein (E:T) of 0.55 to 0.60, as recommended by Heger (2003).

**Table 3:** Composition of experimental diets (%, as is basis).

Ingredients	T1	T2	T3	T4	T5	T6	T7	T8	T9	T10	T11	T12	T13
Wheat	25.29	29.19	27.67	32.00	27.75	24.29	29.94	17.26	30.78	22.62	24.26	25.07	26.93
Barley	27.50	25.00	20.00	25.22	20.00	20.00	20.00	25.00	20.00	18.00	20.00	20.00	20.00
Soybean meal	0.12	0.87	0.06	0.50	8.21	4.55	8.81	9.45	3.26	20.33	20.48	17.90	24.06
Corn	10.00	10.00	16.00	10.00	10.00	13.00	10.00	10.00	10.00	10.00	10.98	10.00	10.61
Sorghum	8.20	10.00	10.00	10.00	5.85	10.00	10.00	5.00	10.00	4.59	5.00	5.00	2.00
Canola meal - solvent extracted	4.00	4.08	0.16	1.01	10.50	4.37	5.57	10.00	7.54	6.00	5.00	6.00	0.10
Wheat pollard	2.00	2.50	0.10	0.50	5.29	2.01	1.27	8.20	0.10	5.00	5.00	5.00	4.98
Canola oil	1.00	2.00	2.07	3.49	1.00	0.51	2.00	6.00	3.83	2.00	2.76	2.72	4.90
Sawdust			2.80	4.00	4.50	4.80	3.00	0.26	0.50	0.50	0.47	0.50	0.70
Rice hulls	5.00	3.67	5.00	0.78	0.80	4.07	1.10	0.20	0.76	4.37	0.50	0.50	0.50
Diatomaceous earth	5.00	2.00	2.00					2.50	2.00	1.50	1.00	1.50	0.33
Bentonite	1.50	1.50	1.50	1.50	1.50	1.50	1.50	1.50	1.50	1.50	1.50	1.50	1.50
Carbohydrases 1	0.01	0.01	0.01	0.01	0.01	0.01	0.01	0.01	0.01	0.01	0.01	0.01	0.01
Phytases 2	0.01	0.01	0.01	0.01	0.01	0.01	0.01	0.01	0.01	0.01	0.01	0.01	0.01
Potassium carbonate	1.00	0.94	1.11	1.03	0.20	0.86	0.50	0.26	0.83	0.25		0.10	0.00
Limestone	1.24	1.25	1.27	1.28	1.15	1.21	1.20	1.15	1.18	1.17	1.19	1.18	1.25
Monocalcium phosphate	0.46	0.42	0.55	0.47	0.26	0.44	0.35	0.27	0.40	0.24	0.22	0.23	0.22
Salt	0.07	0.08	0.04	0.06	0.01	0.12	0.18	0.01	0.01	0.31	0.31	0.12	0.31
Sodium bicarbonate	0.46	0.44	0.51	0.48	0.52	0.38	0.28	0.52	0.53	0.10	0.10	0.37	0.11
TiO_2_	0.50	0.50	0.50	0.50	0.50	0.50	0.50	0.50	0.50	0.50	0.50	0.50	0.50
Vitamins 3	0.07	0.07	0.07	0.07	0.07	0.07	0.07	0.07	0.07	0.07	0.07	0.07	0.07
Trace minerals 3	0.10	0.10	0.10	0.10	0.10	0.10	0.10	0.10	0.10	0.10	0.10	0.10	0.10
Choline chloride, 70%	0.15	0.14	0.16	0.14	0.12	0.14	0.11	0.13	0.13	0.09	0.08	0.09	0.07
L-Lys HCl, 78.4%	0.86	0.83	0.97	0.91	0.46	0.73	0.55	0.43	0.70	0.18	0.18	0.25	0.17
DL-Met	0.27	0.26	0.30	0.28	0.20	0.25	0.22	0.20	0.24	0.21	0.21	0.17	0.17
L-Thr	0.42	0.40	0.48	0.44	0.21	0.36	0.27	0.21	0.33	0.10	0.09	0.12	0.10
L-Trp	0.05	0.04	0.07	0.05		0.03			0.02				
L-Iso	0.49	0.32	0.69	0.51	0.12	0.55	0.30	0.11	0.53	0.10		0.27	0.09
L-Leu	0.59	0.31	0.87	0.60		0.67	0.27		0.63			0.26	
L-Pro	0.60	0.60	1.00	0.85		1.20	0.30		0.90				
L-Cys	0.16	0.15	0.19	0.17	0.07	0.14	0.10	0.08	0.12	0.01		0.06	0.07
L-Ala	0.10	0.10	0.30	0.15		0.05	0.05		0.05				
L-Gly	0.62	0.57	0.71	0.64	0.23	0.50	0.31	0.24	0.45				
L-Arg	0.63	0.59	0.74	0.67	0.24	0.51	0.34	0.22	0.47	0.00		0.06	
L-Val	0.54	0.34	0.77	0.56	0.13	0.63	0.35	0.12	0.59	0.16		0.35	0.16
L-Asp	0.40	0.15	0.15	0.23		0.25	0.15		0.15				
L-Glu	0.60	0.60	1.10	0.80		1.20	0.30		0.80				
Total	100.00	100.00	100.00	100.00	100.00	100.00	100.00	100.00	100.00	100.00	100.00	100.00	100.00
Feed form 4	C	C	C + M	M	C	M + C	M	M	C	M	M	M	M

T = treatment.

1 Carbohydrases (Rovabio Advance T-Flex) included xylanase, β-glucanase and arabinofuranosidase.

2 Phytases (AXTRA PHY, Gold 10T, DuPont Animal Nutrition, Marlborough, UK) provided 500 FTU/kg.

3 Vitamin-mineral concentrate supplied per kilogram of diet: 5040 mg retinol, 17.5 mg cholecalciferol, 105 mg tocopheryl acetate, 4 mg menadione, 4 mg thiamine, 11 mg riboflavin, 77 mg niacin, 18 mg pantothenate, 7 mg pyridoxine, 0.35 mg biotin, 3.0 mg folate, 0.02 mg cyanocobalamin, 23 mg copper, 1.79 mg iodine, 57 mg iron, 171 mg manganese, 0.43 mg selenium and 143 mg zinc.

4 C = crumbles; M = marsh; M + C = mixed mash-crumble.

**Table 4:** Calculated nutrient composition (%, as is basis, unless otherwise indicated).

Nutrient	T1	T2	T3	T4	T5	T6	T7	T8	T9	T10	T11	T12	T13
AME, MJ/kg	11.51	12.34	12.34	13.15	11.57	11.59	12.40	13.11	13.20	11.60	12.43	12.44	13.22
AMEn, MJ/kg	11.35	12.17	12.18	12.99	11.38	11.41	12.22	12.93	13.01	11.39	12.23	12.23	13.02
NE, MJ/kg	9.00	9.70	9.70	10.40	9.00	9.00	9.70	10.40	10.40	9.00	9.70	9.70	10.40
CP	15.00	15.00	15.00	15.00	17.00	17.00	17.00	17.00	17.00	19.00	19.00	19.00	19.00
Crude fat	2.69	3.81	3.75	5.18	2.93	2.28	3.83	7.92	5.62	3.79	4.63	4.56	6.61
Crude fibre	6.52	5.79	7.23	6.06	7.44	8.47	6.14	5.03	4.44	6.92	4.88	4.88	4.63
EAA	7.95	7.53	8.22	7.87	8.26	8.68	8.54	8.28	8.79	9.21	9.01	9.63	9.19
TP	13.65	13.50	14.10	13.90	14.60	15.50	15.30	14.23	15.63	16.39	16.36	16.43	16.41
D. Gly_equi_^1^	1.140	1.140	1.140	1.140	1.140	1.140	1.140	1.140	1.140	1.135	1.146	1.089	1.151
D. Arg	1.075	1.075	1.075	1.075	1.075	1.075	1.075	1.075	1.076	1.075	1.075	1.075	1.075
D. Lys^2^	0.995	0.995	0.995	0.995	0.995	0.995	0.995	0.995	0.995	0.995	0.995	0.995	0.995
D. Met	0.418	0.418	0.423	0.418	0.423	0.418	0.418	0.422	0.418	0.463	0.463	0.418	0.418
D. Met + Cys	0.746	0.746	0.746	0.746	0.746	0.746	0.746	0.746	0.746	0.746	0.746	0.746	0.746
D. Trp	0.169	0.169	0.169	0.169	0.198	0.169	0.179	0.200	0.169	0.238	0.240	0.230	0.239
D. Leu	1.304	1.085	1.518	1.301	1.085	1.518	1.301	1.085	1.518	1.301	1.321	1.518	1.301
D. Ile	0.824	0.687	0.961	0.824	0.687	0.961	0.824	0.687	0.961	0.824	0.730	0.961	0.824
D. Thr	0.697	0.697	0.697	0.697	0.697	0.697	0.697	0.697	0.697	0.697	0.697	0.697	0.697
D. Val	0.955	0.796	1.114	0.955	0.796	1.114	0.955	0.796	1.114	0.955	0.806	1.114	0.955
D. Gly	0.908	0.881	0.934	0.906	0.743	0.847	0.764	0.748	0.830	0.621	0.625	0.597	0.617
D. Ser	0.325	0.363	0.289	0.327	0.556	0.410	0.526	0.549	0.433	0.719	0.730	0.689	0.748
Calcium	0.760	0.760	0.760	0.760	0.760	0.760	0.760	0.760	0.760	0.760	0.760	0.760	0.760
Av. P	0.380	0.380	0.380	0.380	0.380	0.380	0.380	0.380	0.380	0.380	0.380	0.380	0.380
Sodium	0.220	0.220	0.220	0.220	0.220	0.220	0.220	0.220	0.220	0.220	0.220	0.220	0.220
Potassium	0.950	0.950	0.944	0.950	0.755	0.950	0.864	0.809	0.947	0.950	0.820	0.834	0.835
Chloride	0.300	0.300	0.300	0.300	0.180	0.300	0.300	0.180	0.226	0.300	0.300	0.200	0.300
Choline, mg/kg	1500	1500	1500	1500	1500	1500	1500	1500	1500	1500	1500	1500	1500
Linoleic acid	1.06	1.36	1.40	1.72	1.13	1.00	1.40	2.34	1.82	1.38	1.62	1.58	2.13
Leu:Lys ratio	131	109	153	131	109	153	131	109	153	131	133	153	131
Ile:Lys ratio	83	69	97	83	69	97	83	69	97	83	73	97	83
Val:Lys ratio	96	80	112	96	80	112	96	80	112	96	81	112	96
DEB 3	254	254	253	254	238	254	232	252	274	254	221	253	225
EAA:TP 4	0.58	0.56	0.58	0.57	0.57	0.56	0.56	0.58	0.56	0.56	0.55	0.59	0.56

AME = apparent metabolizable energy; AMEn = AME, corrected of nitrogen or N; NE = net energy; CP = crude protein; EAA = essential amino acids; TP = true protein; D. = digestible; Av. = available; DEB = dietary electrolyte balance.

1 Glycine equivalent (%) = Gly (%) + [0.7143 × Ser (%)], where 0.7143 is the ratio of the molar weight between Gly and Ser (Dean et al., 2006).

2 The Lys value of 0.995% was computed as an average of Cobb finisher 1 and finisher 2 (Cobb500, 2018). The remaining amino acids were calculated based on the Lys ratios from Texas A&M (Wu, 2014).

3 DEB (mEq/kg) = Na/0.0023 + K/0.00391 - Cl/0.00355, where Na, K and Cl are in percentages (Mongin, 1981).

4 EAA:TP, ingredient nitrogen (N) × ingredient specific N to protein conversion factor (K_A_) (Alhotan and Pesti, 2016).

**Table 5:** Analyzed ingredient AA (%, as is basis).

Item	Barley	Canola meal	Corn	Wheat pollard	Soybean meal	Sorghum	Wheat
His	0.302	1.092	0.291	0.389	1.272	0.288	0.311
Ser	0.466	1.652	0.384	0.592	2.327	0.472	0.513
Arg	0.528	2.285	0.326	0.774	3.283	0.362	0.508
Gly	0.435	1.966	0.278	0.642	1.958	0.315	0.472
Asp	0.666	2.826	0.560	0.893	5.385	0.715	0.617
Glu	2.865	7.282	1.612	3.326	8.657	2.306	3.440
Thr	0.360	1.645	0.273	0.430	1.821	0.328	0.316
Ala	0.434	1.665	0.599	0.567	1.996	0.965	0.390
Pro	1.270	2.386	0.760	1.157	2.336	0.842	1.081
Lys	0.397	2.134	0.208	0.497	2.874	0.219	0.312
Tyr	0.235	0.876	0.204	0.282	1.375	0.300	0.225
Met	0.153	0.416	0.126	0.170	0.371	0.143	0.148
Val	0.562	2.026	0.384	0.644	2.298	0.536	0.490
Iso	0.413	1.591	0.290	0.475	2.211	0.432	0.397
Leu	0.776	2.730	1.049	0.876	3.567	1.422	0.731
Phe	0.600	1.560	0.401	0.603	2.373	0.551	0.501
Cys	0.248	0.733	0.187	0.298	0.612	0.180	0.232
Trp	0.136	0.523	0.048	0.152	0.571	0.111	0.123

**Table 6:** Analyzed concentrations of CP (%, as is basis), AA (%, as is basis) and energy in treatment diets.

Item	1	2	3	4	5	6	7	8	9	10	11	12	13
His	0.238	0.250	0.200	0.235	0.405	0.293	0.358	0.411	0.318	0.408	0.480	0.445	0.474
Arg	0.921	0.956	0.967	0.997	0.980	1.022	1.005	1.087	1.007	1.041	1.074	0.996	1.056
Thr	0.666	0.671	0.670	0.698	0.725	0.716	0.724	0.774	0.714	0.762	0.754	0.727	0.745
Lys	0.941	0.910	0.926	0.888	0.966	0.985	0.993	1.123	0.987	1.056	1.018	0.969	1.014
Met	0.373	0.348	0.374	0.463	0.352	0.384	0.352	0.440	0.339	0.389	0.468	0.369	0.353
Val	0.932	0.790	1.115	1.001	0.841	1.141	1.002	0.892	1.117	1.107	0.873	1.149	1.020
Ile	0.863	0.674	1.000	0.903	0.711	1.008	0.840	0.743	0.982	0.968	0.762	1.012	0.839
Leu	1.290	1.089	1.554	1.367	1.142	1.578	1.344	1.215	1.552	1.497	1.416	1.544	1.353
Phe	0.436	0.460	0.370	0.443	0.697	0.534	0.649	0.731	0.570	0.727	0.876	0.808	0.886
Cys	0.284	0.288	0.274	0.273	0.349	0.302	0.295	0.356	0.327	0.354	0.342	0.322	0.333
Trp	0.152	0.154	0.141	0.150	0.200	0.142	0.168	0.192	0.156	0.230	0.244	0.236	0.231
Ser	0.416	0.443	0.355	0.418	0.686	0.523	0.639	0.716	0.561	0.727	0.863	0.800	0.868
Gly	1.019	0.922	1.046	1.213	0.881	1.004	0.990	0.963	0.982	0.894	0.768	0.726	0.755
Asp	0.637	0.740	0.585	0.798	1.085	1.044	1.197	1.224	0.991	1.302	1.555	1.374	1.634
Glu	2.767	2.915	2.984	3.156	3.298	3.760	3.391	3.377	3.540	3.715	3.732	3.589	3.751
Ala	0.718	0.564	0.681	0.611	0.658	0.590	0.673	0.704	0.616	0.708	0.805	0.727	0.757
Pro	1.414	1.449	1.710	1.738	1.154	2.104	1.359	1.215	1.837	1.550	1.243	1.210	1.227
Tyr	0.202	0.213	0.179	0.209	0.301	0.270	0.304	0.376	0.257	0.336	0.469	0.359	0.408
BCAA	3.085	2.552	3.670	3.271	2.694	3.727	3.186	2.850	3.651	3.572	3.051	3.706	3.212
CP (16% N)	15.71	15.33	16.17	16.50	17.35	18.10	17.85	18.31	17.56	20.44	19.38	18.65	19.75
NE^1^	9.51	10.58	9.84	10.96	10.16	9.86	10.60	11.09	11.17	9.82	10.60	10.34	11.05
AME^1^	12.72	14.14	13.92	14.92	13.36	13.52	14.00	14.43	14.86	13.25	14.09	13.90	14.66
AMEn^1^	12.10	13.52	13.36	14.25	12.60	12.77	13.20	13.58	14.12	12.35	13.24	13.10	12.10

CP = crude protein; AA = amino acid; NE = net energy; AME = apparent metabolizable energy; N = nitrogen; AMEn = AME corrected of N.

1 AME, AMEn, and NE (MJ/kg DM) were analyzed in closed respiration chambers from d 25 to 28.

All the RP-diets had a similar glycine equivalent. For technical reasons, some diets were pelleted and others were not or partially pelleted. Therefore, birds were offered diets in crumble, mash or mixed mash-crumble form. The feed form (FF) was included in the statistical model during data analysis to count for the FF effect.

### 2.4 Animal management

Birds were brooded and reared based on the Cobb 500 management guidelines (Cobb500, 2022). Day-old as-hatched Cobb 500 broiler chicks obtained from a commercial hatchery (Baiada Poultry Pty Ltd., Tamworth, NSW, Australia) were used in two experiments and they were fed the same diets. These experiments included a floor pen feeding trial and a calorimetric trial. Diet treatments were tested into 7 replicates for the performance trial and 6 times for the calorimetric trial.

In Exp. 1, a total of 1092 birds, 12 birds per pen, was used for the performance trial. Birds and feed were weighed on d 19, 28 and 35, and 4 birds per pen (2 males and 2 females) were sampled on d 35. The sampled birds were randomly selected and euthanized using electrical stunning followed by cervical dislocation to collect ileal digesta contents, abdominal fat pad and breast muscle. In Exp. 2, a total of 156 birds for the calorimetric trial was used and the trial run 6 times using 13 closed respiration chambers, with 2 birds (a male and a female) per chamber. From d 0 to 21, birds were reared in floor pens in a climate-controlled room. They were then acclimatized to the calorimetry chambers from d 21 to 25. The calorimetric run was performed from d 25 to 28 when total excreta was collected daily, birds, feed and O_2_ cylinder weight recorded and respiratory gas exchange measured per chamber on a daily basis for AME and NE analysis.

In both trials, birds were fed diets ad libitum in three phases, including a common starter diet (d 0 to 8), a common grower diet (d 9 to 18) and test finisher diets from d 19 to 35 for Exp. 1, and from d 19 to 28 for Exp. 2. This age group for Exp. 2 was chosen because the NE protocol used in this study was developed using birds aged from d 25 to 28 (Wu et al., 2019).

### 2.5 Chemical analysis and calculations

In Exp. 1, the digesta samples were frozen at −20 °C immediately after collection and then freeze-dried and ground for further analysis. Diet and digesta samples were analyzed for dry matter (DM) by oven-drying at 105 °C until constant weight. The concentrations of AA in ingredients, diets and freeze-dried digesta were analyzed based on the Waters AccQTag AA analysis methodology adapted for use on an Acquity ultra performance liquid chromatography (UPLC) system (Waters Corporation, Milford, MA, USA) (Bosch et al., 2006; Wheat et al., 2008). The TiO_2_ concentration in the diets and digesta samples was analyzed following the protocol described by Short et al. (1996). The apparent ileal digestibility coefficient (dc) was calculated using the equations described by (Adeola, 2001), employing titanium dioxide as an index compound, as follows:

$$\text{dc}=1-\frac{\text{Ti}O_{2}\text{diet}\;\left(\%\right)}{\text{Ti}O_{2}\text{digesta}\;\left(\%\right)}\times \frac{\text{nutrient}\;\text{digesta}\;\left(\%\right)}{\text{nutrient}\;\text{diet}\;\left(\;\%\right)}$$

In Exp. 2, samples for excreta (freeze-dried and ground) and feed were analyzed for gross energy (GE) using an adiabatic bomb calorimeter (Parr 6400 automatic isoperibol calorimeter, Moline, IL, USA) and N content by LECO® FP- 2000 automatic N analyzer (Leco Corporation, St. Joseph, MI, USA). CP was calculated by multiplying the total N obtained from LECO analysis by a standard N conversion factor of 6.25 (Sriperm, 2011).

KOH solution samples were analyzed for CO_2_ recovery following the BaCl_2_ precipitation method described by Annison and White (1961). The volumes (L) of O_2_ consumed and CO_2_ produced at normal temperature and pressure (20 °C and 1 atm) were used to calculate heat production (HP, kcal) based on the modified Brouwer (1965) equation described by Wu et al. (2019) as follows:

$$\text{HP}=1.200\times {\text{CO}}_{2}+3.866\times O_{2}$$

Feed AME (kcal/kg DM) was calculated using the following equation:

$$\text{AME}=\frac{\left[\left(\text{feed}\;\text{GE}\times \text{FI}\right)-\left(\text{excreta}\;\text{GE}\times \text{total}\;\text{excreta}\;\text{output}\right)\right]}{\text{FI}}.$$

Feed NE and NE intake (NEi) were calculated according to Noblet et al. (1994). In short, heat increment was determined by subtracting the fasting (HP) of 450 kJ/kg BW^0.70^ from the total HP. Retained energy was computed by subtracting HP from AME intake (AMEi). NEi was derived from energy retention plus fasting HP multiplied by metabolic BW^0.70^. Dietary NE was then calculated by dividing NEi by FI. The AME and NE values from Exp. 2 were applied to determine AMEi and NEi in Exp. 1.

### 2.6 Statistical analysis

A completely randomized three-level (−1, 0, +1), three-factor (X_1_, X_2_, X_3_) Box-Behnken multivariate design was employed using 13 treatments replicated 7 times for Exp. 1 and 6 times for Exp. 2. All data were analyzed applying RSM in John's Macintosh Project (JMP) 18 Pro (JMP Software, SAS Institute Inc., Cary, NC, 2019) procedure for linear, quadratic, and interactive effects. The percentage of males was used as a covariate for Exp. 1 and the run effect for Exp. 2. Feed form was included in the model for both experiments to count for its effect.

The relationship between the measured responses (Y) and five independent variables (CP, NE, BCAA, male%, and FF) were evaluated by fitting a second-order polynomial mathematical model to the data. The generalized form of equation is as follows:

$$y=\beta_{0}+\sum_{i=1}^{n}\beta_{i}\left(\frac{x_{i}-c_{i}}{d_{i}}\right)+\sum_{n=1}^{n}\sum_{j=i+1}^{n}\beta_{\text{ij}}\left(\;\frac{x_{i}-c_{i}}{d_{i}}\times \frac{x_{j}-c_{j}}{d_{j}}\right)+\sum_{i=1}^{n}\beta_{\text{ii}}{\left(\frac{x_{i}-c_{i}}{d_{i}}\right)}^{n}+\text{Match}\left(\text{FF}\right),$$

where *y* is the measured response; β_0_ is intercept;

$$\sum_{i=1}^{n}\beta_{i}\left(\frac{x_{i}-c_{i}}{d_{i}}\right)\;\text{is}\;a\;\text{linear}\;\text{term;}$$

$$\sum_{n=1}^{n}\;\sum_{j=i+1}^{n}\beta_{\text{ij}}\left(\;\frac{x_{i}-c_{i}}{d_{i}}\times \frac{x_{j}-c_{j}}{d_{j}}\right)\;\text{is}\;\text{an}\;\text{interaction}\;\text{term;}$$

$$\sum_{i=1}^{n}\beta_{\text{ii}}{\left(\frac{x_{i}-c_{i}}{d_{i}}\right)}^{n}\;\text{represents}\;\text{non}-\text{linear}\;\text{effects}\;\text{of}\;\text{the}\;\text{predictors;}$$

Match (FF) accounts for categorical effects.

The experimental unit was a pen mean for Exp. 1 and a chamber mean for Exp. 2, and a 5% level of probability was considered to be significant. The effect of male (%) was adjusted to 50 (50% males and 50% females), whereas the effect of FF was adjusted to 3 (mash) to generate the response surface plots. The mash form was chosen for adjustment due to its representation of more than half (53.8%) of all FF to reduce variations. The non-normally distributed data were transformed using the fitted distribution function of JMP. Interaction plots and response surface plots with contour lines were generated after excluding non-significant (*P* > 0.05) variables from the prediction model. The correlation analysis between the measured responses and the experimental factors (CP, NE, and BCAA) was undertaken using JMP's multivariate correlation analysis, based on the analyzed values of those factors.

## 3 Results

The overall mortality during the experimental period (d 19–35) was less than 3% and there was no dietary treatment-related mortality (*P* > 0.05, data not shown). The design of the independent variables (CP, NE and BCAA) and the corresponding measured bird responses (live performance, carcass quality and nutrient utilization) are presented in Table 7. The experimental data were analyzed and fitted to Standard Least Squares to obtain the regression models.

**Table 7:** Box-Behnken design of three factors (CP, NE and BCAA) along with the mean responses using response surface methodology (RSM) from d 19 to 35.

Independent variables				Experimental responses (dependent variables)							
Treatment	CP, %	NE, MJ/kg	Excess BCAA, %	WG, g	FI, g DM	FCR	AMEi, MJ	NEi, MJ	Lys dc d 35	Breast yield d 35, %	Fat pad d 35, %
1	15	9.0	20	71.9	142	1.98	1812	1354	0.82	5.92	1.26
2	15	9.7	0	82.9	149	1.79	2103	1573	0.78	6.63	1.48
3	15	9.7	40	43.6	103	2.35	1427	1008	0.72	5.45	1.29
4	15	10.4	20	53.8	112	2.08	1670	1227	0.78	5.43	1.39
5	17	9.0	0	94.7	153	1.62	2051	1559	0.74	8.50	1.11
6	17	9.0	40	80.7	145	1.80	1964	1432	0.85	6.69	1.26
7	17	9.7	20	91.0	149	1.64	2086	1579	0.74	8.13	1.26
8	17	10.4	0	93.9	148	1.57	2130	1638	0.78	8.21	1.10
9	17	10.4	40	86.4	139	1.60	2058	1547	0.76	6.57	1.32
10	19	9.0	20	84.0	140	1.67	1861	1379	0.76	8.86	0.94
11	19	9.7	0	88.0	143	1.62	2008	1511	0.76	8.61	1.02
12	19	9.7	40	91.7	147	1.61	2044	1521	0.71	8.05	1.17
13	19	10.4	20	96.4	147	1.53	2156	1625	0.66	8.24	1.21
SEM				1.7	2	0.02	23	19	0.01	0.14	0.02

CP = crude protein; NE = net energy; BCAA = branched-chain amino-acids; WG = weight gain/bird per day; FI = feed intake/bird per day; DM = dry matter; FCR = feed conversion ratio corrected for mortality (g/g DM); AMEi = apparent metabolizable energy intake/bird per day; NEi = net energy intake/bird per day; Lys dc = apparent ileal Lys digestibility coefficient.

The value of regression coefficients and summary statistics for bird responses to the experimental treatments are exhibited in Table 8, Table 9. The lack-of-fit tests, which were not significant *(P* > 0.05), and the residual plots (not presented) for all the response models, indicated that the developed models were well-fitted to the data for predicting and determining the experimental results. The adequacy of the developed models was further validated by the coefficient of determination (*R*^2^), which demonstrated the ability of the models to describe the measurements.

**Table 8:** ANOVA, regression coefficients and summary statistics of growth performance in response to CP, NE and BCAA from d 19 to 35 post–hatch.^1^.

Item	WG		FI		FCR	
	Coefficient	*P*-value	Coefficient	*P*-value	Coefficient	*P*-value
**Linear**						
CP	0.147	<0.001	–	–	−0.308	<0.001
NE	–	–	−0.101	<0.001	−0.143	<0.001
BCAA	−0.007	<0.001	−0.011	<0.001	0.004	<0.001
Male %	0.004	<0.001	0.003	0.032	−0.003	<0.001
CP × male	0.004	<0.001	0.004	0.036	−0.003	<0.001
FF [2-1]	0.330	<0.001	0.505	<0.001	0.065	0.127
FF [3-2]	0.058	0.406	−0.099	0.309	0.031	0.532
**Quadratic**						
CP × CP	−0.246	<0.001	−0.222	<0.001	0.159	<0.001
NE × NE	–	–	–	–	–	–
BCAA × BCAA	0.000	0.039	–	–	–	–
**Interactions**						
CP × NE	0.236	<0.001	0.252	<0.001	−0.120	<0.001
CP × BCAA	0.010	<0.001	0.017	<0.001	−0.003	0.001
NE × BCAA	0.011	<0.001	0.011	<0.001	–	–
Intercept	0.334	<0.001	0.443	<0.001	0.426	<0.001
*R*^2^	0.874		0.674		0.915	
*R*^2^ Adj.	0.856		0.633		0.905	
Lack-of-fit		0.170		0.121		0.477
Model *P*-value		<0.001		<0.001		<0.001

WG = weight gain; FI = feed intake; DM = dry matter; FCR = feed conversion ratio corrected for mortality; CP = crude protein; NE = net energy; BCAA = branched-chain amino-acids; FF = feed form.

1 Linear, quadratic, and interaction regression analyses were generated using the fit linear regression model (standard least squares) procedure of John's Macintosh Project (JMP).

**Table 9:** ANOVA, regression coefficients and summary statistics of and carcass quality and nutrient utilization in response to CP, NE and BCAA from d 19 to 35 post–hatch.^1^

Item	Breast yield		Abdominal fat		Lys dc d35		NEi	
	Coefficient	*P*-value	Coefficient		Coefficient	*P*-value	Coefficient	*P*-value
**Linear**								
CP	–	–	–	–	0.869	<0.001	0.218	<0.001
NE	−0.049	0.004	0.134	<0.001	0.142	<0.001	0.179	<0.001
BCAA	–	–	–	–	−0.093	0.001	−0.011	<0.001
Male %	–	–	–	–	–	–	–	–
FF [2-1]	0.036	0.526	0.117	0.114	−0.562	<0.001	0.160	0.028
FF [3-2]	0.178	<0.001	−0.368	<0.001	0.266	<0.001	−0.344	0.003
**Quadratic**								
CP × CP	–	–	–	–	−0.209	<0.001	−0.262	<0.001
NE × NE	–	–	−0.107	0.023	–	–	−0.116	0.002
BCAA × BCAA	0.000	0.006	0.000	0.003	–	–	0.000	0.009
**Interactions**								
CP × NE	0.105	<0.001	–	–	–	–	0.128	<0.001
CP × BCAA	0.003	0.041	0.006	0.007	−0.004	0.048	0.011	<0.001
NE × BCAA	0.003	0.029	–	–	−0.020	<0.001	–	–
Intercept	0.116	0.003	1.498	<0.001	0.820	<0.001	1.110	<0.001
*R*^2^	0.369		0.337		0.655		0.822	
*R*^2^ Adj.	0.315		0.290		0.624		0.800	
Lack-of-fit		0.261		0.432		0.488		0.291
Model *P*-value		<0.001		<0.001		<0.001		<0.001

Lys dc = apparent ileal Lys digestibility coefficient; NEi = net energy intake/bird per day; CP = crude protein; NE = net energy; BCAA = branched-chain amino-acids; FF = feed form.

1 Linear, quadratic, and interaction regression analyses were generated using the fit linear regression model (standard least squares) procedure of JMP.

A series of interaction plots coupled with three-dimensional response surface graphs with contour plots were constructed for measurements analyzed with *P* < 0.05. Within these graphical representations, one variable was held constant, while the other two underwent variations, facilitating the examination of interactive effects arising from these independent variables on the measured variables. Most of the response surface plots showed peaks, indicating that the optimum points of the measured responses were within the limits of the study design.

### 3.1 Feed intake

**Fig. 1:**
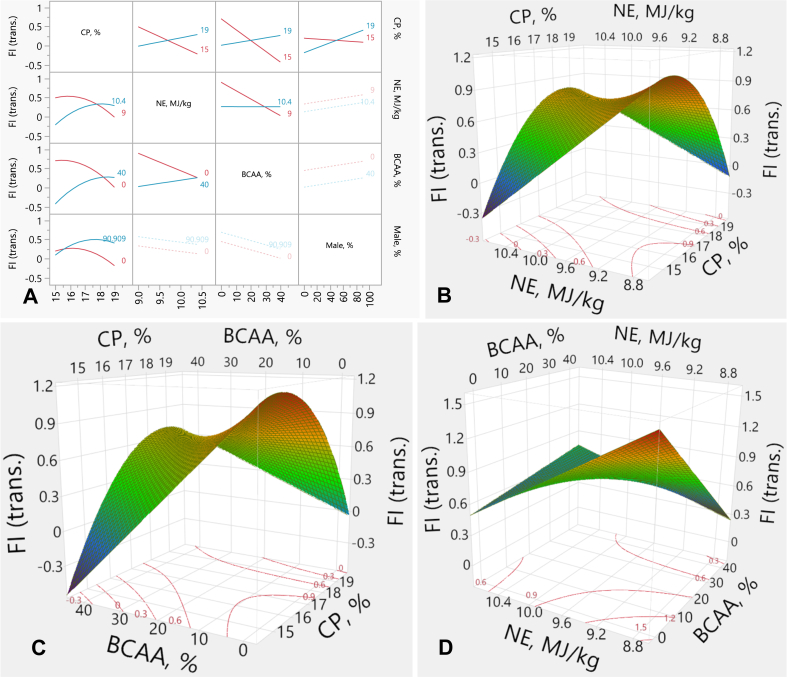
Response surface describing the relationship between FI and dietary CP, NE and BCAA in broilers from d 19 to 35. (A) Interaction plots illustrating the pairwise effects of CP, NE, and BCAA on transformed FI, with percentage of males included as a covariate. (B) Response surface plot showing the interaction between CP and NE on transformed FI, with BCAA held constant at 20%. (C) Response surface plot illustrating the interaction between CP and BCAA on transformed FI, with NE held constant at 9.7 MJ/kg. (D) Response surface depicting the interaction between NE and BCAA on transformed FI, with CP held constant at 17%. FI = feed intake; trans. = transformed values; CP = crude protein; NE = net energy; BCAA = branched-chain amino-acids.

There was a negative linear effect (*P* < 0.001) between NE and FI and between BCAA and FI. CP showed a quadratic effect (*P* < 0.001) on FI (Table 8). Interactive effects (*P* < 0.001) between all experimental factors (CP, NE, BCAA) on FI were observed (Fig. 1). NE had no effect on FI in high CP (HCP)-diets, however, reducing NE in the RP-diets led to higher FI than higher NE (Fig. 1A and B). BCAA levels had no effect on FI in HCP-diets. In the RP-diets, however, increasing BCAA led to much lower FI than the lower BCAA content (Fig. 1A and C). The effect of BCAA on FI was not significant in high-NE diets. However, increasing BCAA in the low-NE diets led to a depressed FI compared with the lower BCAA content (Fig. 1A and D).

The FI response (*y*) was described by the following equation from the transformed data:

$$y=\;0.4428+\frac{\left(\frac{x_{1}-17}{2}\right)\left(x_{1}-17\right)}{2}\times \left(-0.2224\right)+\frac{\left(\frac{x_{1}-17}{2}\right)\left(x_{2}-9.7\right)}{0.7}\times 0.2521+\left(-0.1012\right)\times \frac{x_{2}-9.7}{0.7}+\left(-0.0108x_{3}\right)+\frac{x_{1}-17}{2}\times \left(x_{3}-20\right)\times 0.0173+\frac{x_{2}-9.7}{0.7}\times \left(x_{3}-20\right)\times 0.0108+\frac{x_{1}-17}{2}\times \left(m-49.64\right)0.0038+0.0027m+\text{Match}\left(\text{FF}\right)\times \left\{ \begin{array}{c} 1\Rightarrow 0.000\;\\ 2\Rightarrow 0.5053\\ 3\Rightarrow 0.4060\\ else\Rightarrow 0.000 \end{array}\right.$$

where *x*_*1*_ is CP concentration (%), *x*_*2*_ is NE density (MJ/kg), *x*_*3*_ is BCAA above requirements (%), *m* is male %, FF is feed form (1: crumbled, 2: mixed mash-crumbled, 3: mash).

FI (*y*) transformation equation equals to:

$$\text{Normal}\;\text{Mixture}\;\text{Distribution}\;\left(y,\left[106.2,145.4\right],\left[5.481,6.629\right],\left[0.145,0.855\right]\right)$$

### 3.2 Weight gain

**Fig. 2:**
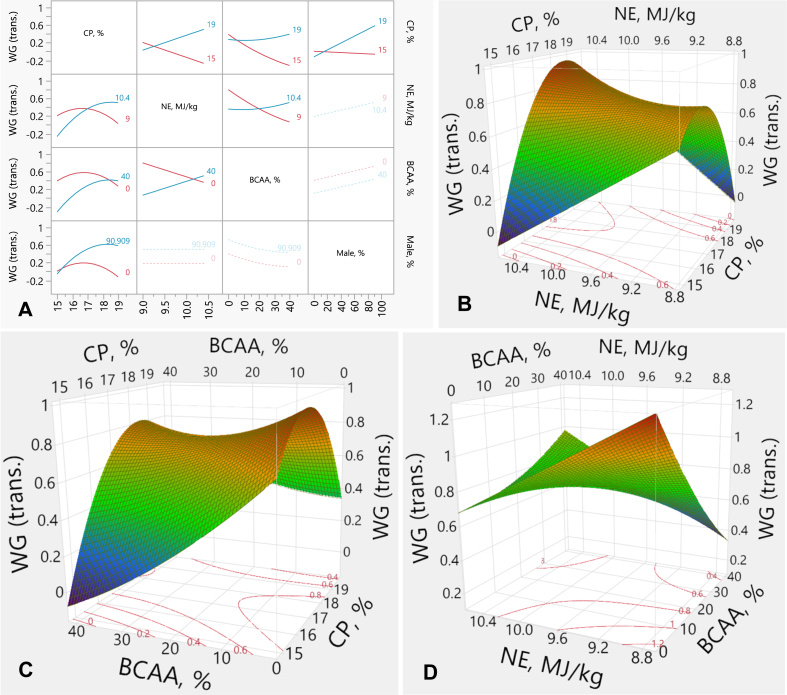
Response surface describing the interactive effects between WG and dietary CP, NE and BCAA in broilers from d 19 to 35. (A) Interaction plots showing the pairwise effects of CP, NE, and BCAA on transformed WG, with percentage of males included as a covariate. (B) Response surface plot illustrating the interaction between CP and NE on transformed WG, with BCAA held constant at 20%. (C) Response surface plot depicting the interaction between CP and BCAA on transformed WG, with NE held constant at 9.7 MJ/kg. (D) Response surface illustrating the interaction between NE and BCAA on transformed WG, with CP held constant at 17%. WG = weight gain; trans. = transformed values; CP = crude protein; NE = net energy; BCAA = branched-chain amino-acids.

The interaction plots between the factors and WG and their corresponding three-dimensional response surface graphs are shown in Fig. 2. There was a linear effect of CP and BCAA on WG (*P* < 0.001, each), a quadratic effect of CP (*P* < 0.001) and BCAA (*P* = 0.039) on WG, and interactive effects (*P* < 0.001) between CP and NE, CP and BCAA, and NE and BCAA on WG. The effect of CP on WG was similar at the lower level of NE. However, at the high level of NE, reducing CP led to a lower WG than the higher CP content (Fig. 2A and B). Additionally, the effect of BCAA on WG did not differ in the HCP-diets. However, increasing BCAA % in the RP-diets resulted in much lower WG than the lower BCAA content (Fig. 2A and C). The interactive effect between NE and BCAA on WG shows that the effect of BCAA on WG was the same at the high level of NE. However, increasing BCAA in the low NE-diets, led to a lower WG than the low BCAA content (Fig. 2A and D). The WG response (*y*) was predicted by the model equation (from the transformed data):

$$y=0.3337+0.1474\times \frac{x_{1}-17}{2}+\frac{\left(\frac{x_{1}-17}{2}\right)\left(x_{2}-9.7\right)}{0.7}\times 0.2356+\frac{x_{1}-17}{2}\times \left(x_{3}-20\right)\times 0.0103+\frac{x_{2}-9.7}{0.7}\times \left(x_{3}-20\right)\times 0.0110+\left(-0.0073x_{3}\right)+0.0035m+\frac{\left(\frac{x_{1}-17}{2}\right)\left(x_{1}-17\right)}{2},\times \left(-0.2463\right)+\left(x_{3}-20\right)\left(x_{3}-20\right)0.0002+\frac{x_{1}-17}{2}\times \left(m-49.64\right)\times 0.0042+\text{Match}\left(\text{FF}\right)\times \left\{ \begin{array}{c} 1\Rightarrow 0.000\;\\ 2\Rightarrow 0.3301\\ 3\Rightarrow 0.3881\\ else\;0.000 \end{array}\right.$$

where *x*_*1*_ is CP concentration (%), *x*_*2*_ is NE density (MJ/kg), *x*_*3*_ is BCAA above requirements (%), *m* is male %, FF is feed form (1: crumbled, 2: mixed mash-crumbled, 3: mash). The transformation equation equals:

NormalMixtureDistribution(y,[48.73,71.56,89.22],[5.044,2.548,5.985],[0.1539,0.0863,0.7598])

### 3.3 Feed conversion ratio

**Fig. 3:**
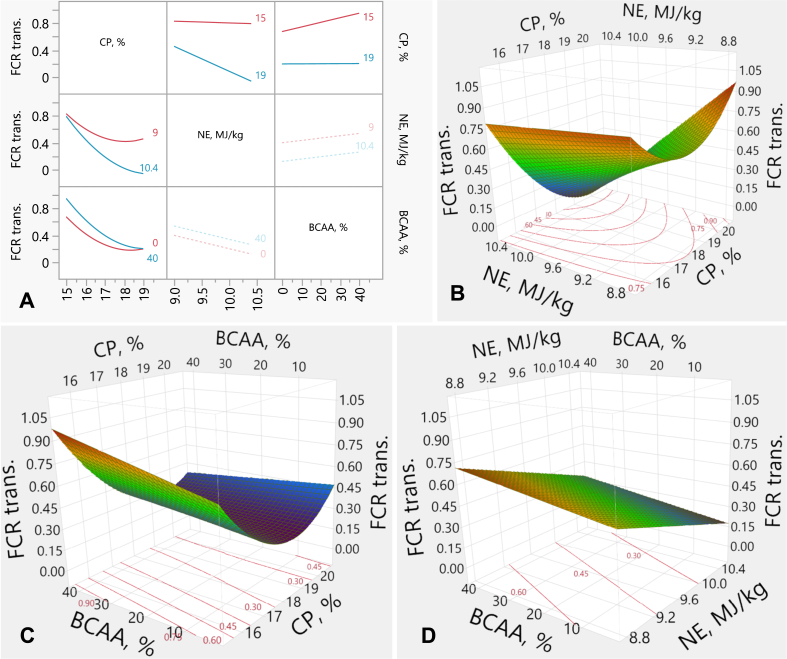
Response surface illustrating the interactive effects between FCR and dietary CP, NE and BCAA in broilers from d 19 to 35. (A) Interaction plots showing the pairwise effects of CP, NE, and BCAA on transformed FCR. (B) Response surface plot illustrating the interaction between CP and NE on transformed FCR, with BCAA held constant at 20%. (C) Response surface plot showing the interaction between CP and BCAA on transformed FCR, with NE held constant at 9.7 MJ/kg. (D) Response surface describing the interaction between NE and BCAA on transformed FCR, with CP held constant at 17%. Where, FCR = feed conversion ratio corrected for mortality (g/g dry matter basis); trans. = transformed values; CP = crude protein; NE = net energy; BCAA = branched-chain amino-acids.

The experimental factors (CP, NE and BCAA) had significant *(P* < 0.001, each) linear effects on FCR. In addition, CP had a quadratic effect (*P* < 0.001) on FCR. The interactive effects of CP by NE (*P* < 0.001), and CP by BCAA (*P* = 0.001) on FCR were also observed (Fig. 3A–C). NE showed no effect on FCR in the RP-diets, whereas increasing NE in the HCP-diets decreased FCR (Fig. 3A and B). Moreover, the effect of BCAA on FCR did not differ in the HCP-diets. However, increasing BCAA % in the RP-diets increased FCR (Fig. 3A and C). There was no interaction *(P* > 0.05) of NE by BCAA on FCR (Fig. 3A and D). FCR increased with reducing NE contents, irrespective of BCAA levels, and FCR reduced with dietary BCAA, irrespective of NE levels. The response of FCR (*y*) was described by the following equation from the transformed values,

$$y=0.4256+\left(-0.3079\right)\times \frac{x_{1}-17}{2}+\left(-0.1431\right)\times \frac{x_{2}-9.7}{0.7}+0.0036x_{3}+\left(-0.0033m\right)+\frac{\left(\frac{x_{1}-17}{2}\right)\left(x_{1}-17\right)}{2}\times 0.1588+\frac{\left(\frac{x_{1}-17}{2}\right)\left(x_{2}-9.7\right)}{0.7}\times \left(-0.1195\right)+\frac{x_{1}-17}{2}\times \left(x_{3}-20\right)\left(-0.0034\right)+\frac{x_{1}-17}{2}\times \left(m-49.64\right)(-0.0033+\text{Match}\left(\text{FF}\right)\times \left\{ \begin{array}{c} 1\Rightarrow 0.000\;\\ 2\Rightarrow 0.0650\\ 3\Rightarrow 0.0960\\ else\Rightarrow 0.000 \end{array}\right.$$

where *x*_*1*_ is CP concentration (%), *x*_*2*_ is NE density (MJ/kg), *x*_*3*_ is BCAA above requirements (%), *m* is male %, FF is feed form (1: crumbled, 2: mixed mash-crumbled, 3: mash). The transformation equation equals to:

NormalMixtureDistribution(y,[1.607,1.906,2.381],[0.0519,0.1386,0.0339],[0.5935,0.3408,0.0656])

### 3.4 Breast yield

**Fig. 4:**
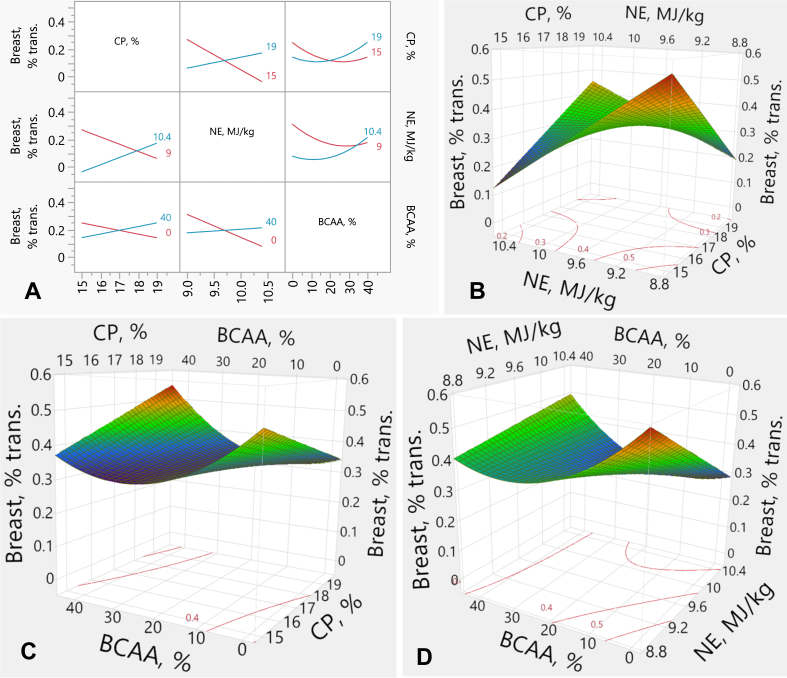
Response surface describing interactions between breast yield and dietary CP, NE and BCAA in broilers on d 35. (A) Interaction plots showing the pairwise effects of CP, NE, and BCAA on transformed breast yield. (B) Response surface plot illustrating the interaction between CP and NE on transformed breast yield, with BCAA held constant at 20%. (C) Response surface plot showing the interaction between CP and BCAA on transformed breast yield, with NE held constant at 9.7 MJ/kg. (D) Response surface describing the interaction between NE and BCAA on transformed breast yield, with CP held constant at 17%. Where, trans. = transformed values; CP = crude protein; NE = net energy; BCAA = branched-chain amino-acids.

The interactive effects, interaction plots and response surface with contour plots for breast yield are presented in Table 9 and Fig. 4. There was a linear effect (*P* = 0.004) between NE and breast yield. BCAA also had a quadratic effect (*P* = 0.006) on the yield. All factors had significant interactive effects on breast rate: CP × NE (*P* < 0.001), CP × BCAA (*P* = 0.041), and NE × BCAA (*P* = 0.029). The effect of NE on breast yield was similar in HCP-diets. However, the lower energy density in RP-diets resulted in a better breast yield than the higher energy density (Fig. 4A and B). In addition, high BCAA % in the RP-diets depressed breast yield relative to low BCAA %. Conversely, high BCAA % in the HCP-diets increased the yield compared to low BCAA % (Fig. 4A and C). Moreover, NE levels did not influence the breast yield in high-BCAA diets. However, at the lower BCAA content, the low NE density increased breast yield compared to the higher energy density (Fig. 4A and D). Breast yield (*y*) was predicted from the transformed values by the following equation:

$$y=0.1161+\frac{\left(\frac{x_{1}-17}{2}\right)\left(x_{2}-9.7\right)}{0.7}\times 0.1048+\frac{x_{1}-17}{2}\times \left(x_{3}-20\right)\times 0.0027+\frac{x_{2}-9.7}{0.7}\times \left(x_{3}-20\right)0.0033+\left(x_{3}-20\right)\left(x_{3}-20\right)0.0002+\left(-0.0492\right)\times \frac{x_{2}-9.7}{0.7}+\text{Match}\left(\text{FF}\right)\times \left\{ \begin{array}{c} 1\Rightarrow 0.0000\;\\ 2\Rightarrow 0.0363\\ 3\Rightarrow 0.2145\\ else\;\Rightarrow 0.000 \end{array}\right.$$

where *x*_*1*_ is CP concentration (%), *x*_*2*_ is NE density (MJ/kg), *x*_*3*_ is BCAA above requirements (%). The breast (*y*) transformation function equals:

NormalMixtureDistribution(y,[5.979,8.362],[0.5324,0.4653],[0.4350,0.5650])

### 3.5 Fat pad

**Fig. 5:**
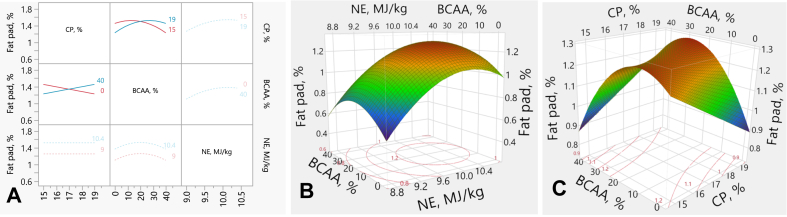
Response surface describing interactions between abdominal fat pad and dietary CP, NE and BCAA in broilers on d 35. (A) Interaction plots showing the pairwise effects of CP, NE, and BCAA on fat pad. (B) Response surface plot showing the quadratic effects of NE and BCAA on fat pad, with CP held constant at 17%. (C) Response surface plot illustrating the interaction between CP and BCAA on fat pad, with NE held constant at 9.7 MJ/kg. CP = crude protein; NE = net energy; BCAA = branched-chain amino-acids.

The interaction plots and response surface plots for the abdominal fat pad rate are illustrated in Fig. 5. There was a linear effect (*P* < 0.001) between NE and fat pad, showing that the fat pad increased with dietary NE, irrespective of CP or BCAA. The negative quadratic effect (*P* = 0.023) was observed between NE and fat pad, and a positive quadratic effect (*P* = 0.003) was observed between BCAA and fat (Fig. 5B). BCAA also interacted with CP (*P* = 0.007) on the fat pad, where higher BCAA % led to lower fat than the lower BCAA % in the RP-diets. In the HCP-diets, however, higher BCAA % resulted in a higher fat than the low BCAA content (Fig. 5 A and C). There was no interactive effect between CP and NE or between NE and BCAA on fat accretion. The fat pad rate (*y*) was predicted by the following equation:

$$y=1.498+\frac{x_{1}-17}{2}\times \left(x_{3}-20\right)\times 0.0057+0.1338\times \frac{x_{2}-9.7}{0.7}+\frac{\left(\frac{x_{2}-9.7}{0.7}\right)\left(x_{2}-9.7\right)}{0.7}\times (-0.1069+\left(x_{3}-20\right)\left(x_{3}-20\right)\left(-0.0004\right)+\text{Match}\left(\text{FF}\right)\times \left\{ \begin{array}{c} 1\Rightarrow 0.0000\;\\ 2\Rightarrow 0.1173\\ 3\Rightarrow -0.2508\\ else\;\Rightarrow 0.000 \end{array}\right.$$

where *x*_*1*_ is CP concentration (%), *x*_*2*_ is NE density (MJ/kg), *x*_*3*_ is BCAA above requirements (%).

### 3.6 Lysine digestibility coefficient

**Fig. 6:**
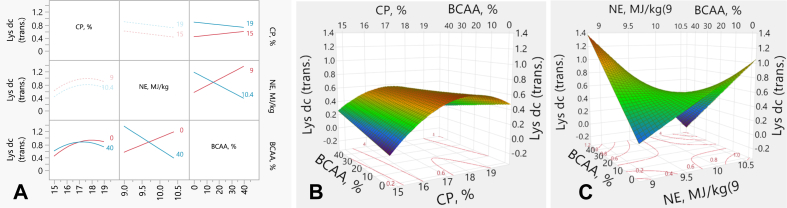
Response surface describing interactions between Lys dc and dietary CP, NE and BCAA in broilers on d 35. (A) Interaction plots showing the pairwise effects of CP, NE, and BCAA on Lys dc. (B) Response surface plot illustrating the interactive effects of CP and BCAA on Lys dc, with NE held constant at 9.7 MJ/kg. (C) Response surface plot showing the interaction between NE and BCAA on Lys dc, with CP held constant at 17%. Lys dc = apparent ileal lysine digestibility coefficient; trans. = transformed values; CP = crude protein; NE = net energy; BCAA = branched-chain amino-acids.

The interaction plots and response surface plots for the apparent ileal Lys dc are illustrated in Fig. 6. The experimental factors (CP, NE and BCAA) had significant (*P* ≤ 0.001) linear effects on Lys dc. There were CP by BCAA (*P* = 0.048), and NE by BCAA (*P* < 0.001) interactive effects on Lys dc. There was no CP effect on Lys dc in the high BCAA-diets. However, Lys dc decreased with CP in the low BCAA diets (Fig. 6A and B). Moreover, Lys dc increased with BCAA in the low-NE diets. However, Lys dc lowered with increased BCAA in the high-NE diets (Fig. 6A and C). There was no interactive effect (*P* > 0.05) between CP and NE; reducing CP content decreased Lys dc, regardless of NE levels; and reducing NE density increased Lys dc, irrespective of CP levels (Fig. 6A). The Lys dc (*y*) was predicted from the transformed values by the following equation:

$$y=0.8693+0.1420\times \frac{x_{1}-17}{2}+\left(-0.0929\;\right)\times \frac{x_{2}-9.7}{0.7}+\frac{\left(\frac{x_{1}-17}{2}\right)\left(x_{1}-17\right)}{2}\times (-0.2087+\frac{x_{1}-17}{2}\times \left(x_{3}-20.23\right)\left(-0.0040\right)+\frac{x_{2}-9.7}{0.7}\times \left(x_{3}-20.23\right)\left(-0.0200\right)+\text{Match}\left(\text{FF}\right)\times \left\{ \begin{array}{c} 1\Rightarrow 0.0000\;\\ 2\Rightarrow -0.5616\\ 3\Rightarrow -0.2959\\ else\;\Rightarrow 0.000 \end{array}\right.$$

where *x*_*1*_ is CP concentration (%), *x*_*2*_ is NE density (MJ/kg), *x*_*3*_ is BCAA above requirements (%).

The transformation formula is:

$$\text{SHASH}\;\text{Distribution}\;\left(y,4.538,1.966,1.768,0.1007\right)$$

### 3.7 Net energy intake

**Fig. 7:**
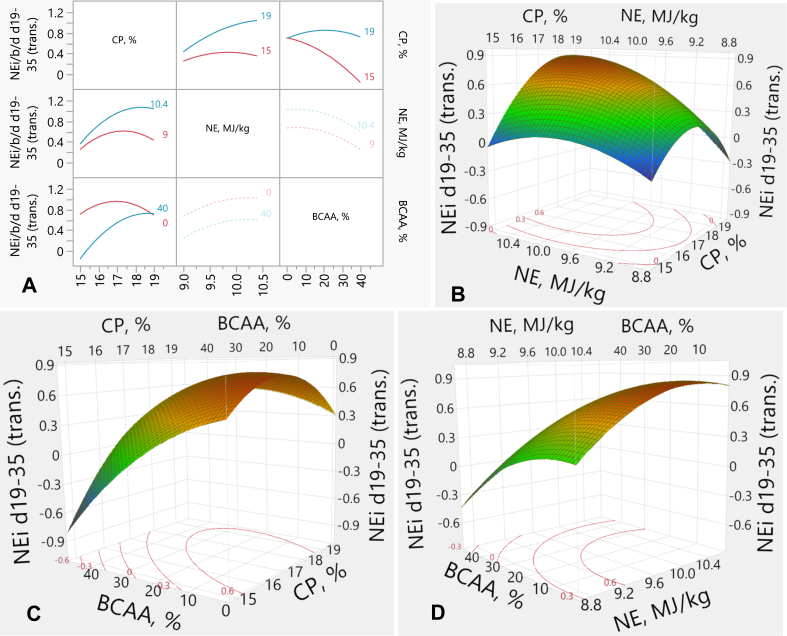
Response surface describing the effects of dietary CP, NE and BCAA on NEi in broilers from d 19 to 35. (A) Interaction plots showing the pairwise effects of CP, NE, and BCAA on transformed NEi. (B) Response surface plot illustrating the interaction between CP and NE on transformed NEi, with BCAA held constant at 20%. (C) Response surface plot showing the interaction between CP and BCAA on transformed NEi, with NE held constant at 9.7 MJ/kg. (D) Response surface describing the interaction between NE and BCAA on transformed NEi, with CP held constant at 17%. Where, NEi = net energy intake; trans. = transformed values; CP = crude protein; NE = net energy; BCAA = branched-chain amino-acids.

The interaction plots and response surface plots for NEi are exhibited in Fig. 7. The experimental factors (CP, NE and BCAA) exhibited significant linear effects on NEi (*P* < 0.001, each), as well as quadratic effects (*P* < 0.001 for CP, *P* = 0.002 for NE, and *P* = 0.009 for BCAA) on NEi. There were interactive effects (*P* < 0.001) between CP and NE and between CP and BCAA on NEi (Fig. 7A). The effect of NE on NEi was similar in birds fed RP-diets. However, in those fed the HCP-diets, high NE increased NEi relative to the low NE content (Fig. 7A and B). In addition, the impact of BCAA on NEi remained similar in the HCP-diets. However, in the RP-diets, high BCAA led to much lower NEi compared to the low BCAA % (Fig. 7A and C). There was no interaction between NE and BCAA on NEi; elevating BCAA levels resulted in decreased NEi irrespective of NE levels, while high NE increased NEi, regardless of BCAA levels (Fig. 7A and D). The response of NEi (*y*) was described from the transformed values by the following equation:

$$y=1.110+0.2183\times \frac{x_{1}-17}{2}+0.1790\times \frac{x_{2}-9.7}{0.7}+\left(-0.0105x_{3}\right)+\frac{\left(\frac{x_{1}-17}{2}\right)\left(x_{1}-17\right)}{2}\times \left(-0.2621\right)+\frac{\left(\frac{x_{1}-17}{2}\right)\left(x_{2}-9.7\right)}{0.7}\times 0.1277+\frac{\left(\frac{x_{2}-9.7}{0.7}\right)\left(x_{2}-9.7\right)}{0.7}\times \left(-0.1155\right)+\frac{x_{1}-17}{2}\times \left(x_{3}-20\right)0.0113+\left(x_{3}-20\right)\left(x_{3}-20\right)\left(-0.0004\right)+\text{Match}\left(\text{FF}\right)\times \left\{ \begin{array}{c} 1\Rightarrow 0.0000\;\\ 2\Rightarrow 0.1596\\ 3\Rightarrow -0.1596\\ else\Rightarrow 0.000 \end{array}\right.$$

where *x*_*1*_ is CP concentration (%), *x*_*2*_ is NE density (MJ/kg), *x*_*3*_ is BCAA above requirements (%). The transformation function equals:

$$\text{Normal}\;\text{Mixture}\;\text{Distribution}\;\left(y,\left[1341,1569\right],\left[181.6,47.45\right],\left[0.4871,0.5129\right]\right)$$

### 3.8 Correlations between experimental independent and dependent variables

Table 10 represents the correlations between factors and the measured responses. WG was correlated with NEi (*r* = 0.946, *P* < 0.001), the measured dietary CP% (*r* = 0.540, *P* < 0.001), and the measured dietary NE (*r* = 0.317, *P* = 0.002), but not with dietary AME (*P* > 0.05). WG was also negatively correlated with BCAA (*r* = −0.298, *P* = 0.004), and fat pad (*r* = −0.239, *P* = 0.022). In addition, BCAA was negatively correlated with FI (*r* = −0.385, *P* < 0.001), dietary NE (*r* = −0.224, *P* = 0.033), NEi (*r* = −0.428, *P* < 0.001), and positively correlated with FCR (*r* = 0.214, *P* = 0.042). Dietary NE was positively correlated with NEi and AMEi (*r* = 0.459 and *r* = 0.483, respectively; *P* < 0.001), and negatively correlated with dietary BCAA (*r* = −0.224, *P* = 0.033). In contrast, dietary AME showed no significant correlation with these variables (*P* > 0.05). In addition, the dietary NE contents were not correlated (*P* > 0.05) with FI nor fat pad content, while dietary AME was correlated with them (*r* = −0.241, *P* = 0.022, and *r* = 0.222, *P* = 0.034, respectively).

**Table 10:** Correlation between the experimental factors and response variables (d 19-35) 1.

Measurement	WG	FI	FCR	MEi	NEi	Breast	Fat pad	CP	BCAA	AME
FI	0.927									
	∗∗∗									
FCR	−0.958	−0.806								
	∗∗∗	∗∗∗								
MEi	0.942	0.918	−0.878							
	∗∗∗	∗∗∗	∗∗∗							
NEi	0.946	0.912	−0.890	0.995						
	∗∗∗	∗∗∗	∗∗∗	∗∗∗						
Breast	0.772	0.634	−0.778	0.605	0.621					
	∗∗∗	∗∗∗	∗∗∗	∗∗∗	∗∗∗					
Fat pad	−0.239	−0.131	0.283	−0.049	−0.070	−0.434				
	∗		∗∗			∗∗∗				
CP	0.540	0.320	−0.640	0.357	0.353	0.776	−0.488			
	∗∗∗	∗∗	∗∗∗	∗∗∗	∗∗∗	∗∗∗	∗∗∗			
BCAA	−0.298	−0.385	0.214	−0.377	−0.428	−0.177	−0.051	0.307		
	∗∗	∗∗∗	∗	∗∗∗	∗∗∗			∗∗		
AME	−0.007	−0.241	−0.139	0.161	0.166	−0.111	0.222	0.064	0.028	
		∗					∗			
NE	0.317	0.085	−0.444	0.459	0.483	0.141	0.124	0.166	−0.224	0.920
	∗∗		∗∗∗	∗∗∗	∗∗∗				∗	∗∗∗

WG = weight gain FI = feed intake; DM = dry matter; FCR = feed conversion ratio corrected for mortality; AMEi = apparent metabolizable energy intake; NEi = net energy intake; Breast = relative breast yield; Fat pad = relative fat pad weight; CP = crude protein; BCAA = branched-chain amino-acids (Leu, Ile and Val); AME = apparent metabolizable energy; NE = net energy.

1 Significant probability values are indicated as follows: ∗*P* < 0.05, ∗∗*P* < 0.01, and ∗∗∗*P* < 0.001.

## 4 Discussion

With continuous improvement in genetics, it is important to determine the optimum levels of digestible AA along with energy densities in diets. BCAA account for 35% to 40% of EAA requirements in poultry (Chrystal et al., 2022; Ospina-Rojas et al., 2017). In addition, BCAA exert important roles in energy homeostasis and regulation of protein and lipid metabolism. Hence, it is crucial to closely control the BCAA dosage, as individual BCAA levels that significantly deviate from the recommendation may negatively impact cell growth and metabolism may occur (Bai et al., 2015; Chrystal et al., 2022; Maynard et al., 2021). To avoid the antagonism of BCAA, the Leu:Ile and Leu:Val ratios were fixed across all the diet treatments in the current study. The study performed a multivariate BBD assessment to compare and visualize the relationships of influential factors based on its statistical techniques that map all possible combinations of the factors while circumventing potential confounding factors (De Leon et al., 2010; Maynard et al., 2021).

The overall WG of the mixed-sexed birds fed HCP-diets from d 19 to 35 (19% CP, treatment 10 to treatment 13) in the present study was above the breeder specifications (90.01 vs 88.00 g/b/d). However, the average WG in birds fed the RP-diets (15% CP, treatment 1 to treatment 4) was inferior to the breed standards (63.04 vs 88.00 g/b/d, Cobb 500 2018), due mainly to the nature of the experimental treatments (low CP, energy and BCAA effects). Due to this variation among the treatment groups, not all the measured variables followed a normal distribution. Therefore, all data except for the fat pad, which was normally distributed, were transformed before data analysis. It is also important to note that the correlation analysis demonstrated contrasting trends of the dietary factors on the measurements compared to the RSM data analysis. This was likely because, unlike the correlation analysis, the RSM data analysis took into account confounding factors (the FF and male percentage effects).

### 4.1 Growth performance

The results from the current study demonstrated that by holding a constant NE density, CP and BCAA interacted to drive bird growth performance (FI, WG and FCR), where BCAA levels had no impact on FI, WG and FCR in the HCP-diets, however increasing BCAA levels in the RP-diets led to a sharp decrease in feed consumption, resulting in a depressed rate of gain and increased FCR. The findings partly agree with a recent study on fast-growing broilers by Zeitz et al. (2019b). The study evaluated the effects of increasing the dietary Leu level by 35% and 60% beyond the recommended amounts in two studies with standard CP-diets. In the first study, they kept the Ile:Val ratio constant, while in the second study, they raised the levels of Ile and Val along with Leu. The findings were consistent: an increase in dietary Leu had no impact on broiler performance. Greenhalgh et al. (2022) also found that elevating BCAA inclusion in a 19% CP-diets did not advantage bird growth rate.

In addition, FI was influenced by the interaction between BCAA and NE in the current study, when the CP level was held constant. BCAA levels had no impact on FI and WG in high-NE diets, but high BCAA depressed the growth performance in low-NE diets. These results are in contrast to the findings of Ospina-Rojas et al. (2017), who investigated the responses of BCAA in broilers offered RP-diets with 16% CP. They observed that the digestible Leu requirements fall within a range of 1.15% to 1.19%, with 0.86% digestible Val needed for optimum WG, and minimum FCR and abdominal fat in RP-diets. However, in the present study, optimal growth performance was achieved with 0% BCAA above requirements, corresponding to 1.085% Leu, 0.720% Ile and 0.796% Val, with 0.995% Lys.

The NE and CP levels also showed an interaction on FI in the present study. NE densities in HCP-diets had no effect on FI, whereas diluting NE from 10.4 to 9.0 MJ/kg in RP-diets increased FI. This is in accordance with the literature, which shows that reducing CP, while maintaining a constant dietary AME or NE content, is associated with a depressed FI due to the high energy-to-CP ratio (Musigwa et al., 2021b; Nawaz et al., 2006). However, the effect of dietary energy on FI has been questioned, suggesting that there are other factors (such as first limiting nutrient and AA densities) driving FI in modern broiler genetics other than the dietary energy (Classen, 2017) or that the FI regulation involves an interaction between dietary energy concentrations and other factors, such as available P and AA (Liu et al., 2019; Sharma et al., 2018). It is important to note that in the RP-diets, the low BCAA level improved FI, leading to optimal WG. This observation was further substantiated by a negative correlation between BCAA and both FI and WG. However, the enhanced FI resulting from NE dilution in the RP-diets only removed the difference in WG between the HCP- and RP-diets. This agrees with the findings of Leeson et al. (1996) that broilers adjust energy consumption by increasing FI when provided with lower-energy diets. In contrast, data from Chrystal et al. (2020) demonstrate that WG increased with the dietary energy content in birds fed diets containing 15.6% CP with 2,870 or 2,971 kcal/kg AME, with no influence of dietary AME on FI.

Conversely, increasing the level of BCAA above the standards in RP-diets led to depressed FI and, consequently, impaired growth performance due to adverse effects on NEi. Similarly, Greenhalgh et al. (2022) observed depressed energy utilization with elevated dietary Leu levels. A surplus in AA supply, specifically BCAA in the current study, may have led to the catabolism of the excess AA, which incurs an energy cost for the elimination of N surplus (Greenhalgh et al., 2022; Heger, 2003; Sklan and Noy, 2004). This potentially explains the depression in energy utilization, which is attributed to the rising levels of BCAA in RP-diets observed in the present study, due to AA antagonism. This will be explained later. The depressed NE utilization caused by excess BCAA was further demonstrated by the observed inverse relationship between dietary BCAA and NE. It has been stated that achieving optimal protein turnover requires a balance between protein and energy. However, to ensure optimal AA utilization for protein synthesis, it is crucial to maintain a sufficient level of feed energy (Maharjan et al., 2021, 2020). Therefore, the observed depressed NEi in the current study not only reduces energy utilization through the AA catabolic process but also leaves insufficient energy for protein synthesis. This, in turn, elucidates the observed poor growth rate in birds fed the RP-diets with elevated BCAA inclusions.

Additionally, the CP by NE interaction was observed for WG in the current study. CP levels had no effect on WG in the low-NE diets, but reducing CP in the high-NE diets depressed WG compared to high CP contents. These findings demonstrated the need to reduce energy in RP-diets, as explained in the literature, that broilers fed RP-diets utilize dietary protein and energy more efficiently (Aletor et al., 2000). It was also discussed that the poor performance of broilers fed HCP-diets could be the result of the deficiency in dietary energy required to process the high dietary protein contents (Gous et al., 2018). The NE and CP levels also interacted to drive different breast meat yields, where NE levels in HCP-diets had no influence on the yield, whereas increasing NE in RP-diets reduced the yield. The decrease in breast yield associated with high NE densities validates what Sharma et al. (2018) and Dozier et al. (2006) observed and is most likely attributed to a depressed FI and, consequently, N intake. However, Liu et al. (2019) suggested that WG and breast meat were influenced by AA levels rather than energy densities. In the present study, a transition from 0% to 40% excess BCAA improved the breast muscle accretion in the HCP-diets, whereas increasing BCAA % in the RP-diets depressed the breast yield. In contrast, Zeitz et al. (2019a) observed that excess dietary Leu concentrations did not influence muscle growth in broilers fed 18% CP-diets at fixed Ile-to-Val ratios. Kidd et al. (2021b) also did not find BCAA effect on breast meat yield response in male broilers fed 19% CP-diets.

### 4.2 Nutrient utilization and carcass quality

A BCAA by NE interaction indicated that increasing BCAA elevated Lys dc in low NE-diets and eliminated the difference in Lys dc between RP- and HCP-diets. This might be linked to the depressive effect of excess BCAA on feed consumption. It was previously observed that under suboptimal N intake, less N is excreted because the majority of it is utilized, thereby improving its efficiency (Bregendahl et al., 2002; Musigwa et al., 2021b, 2020; Widyaratne and Drew, 2011). This suggests that birds with depressed FI consumed less Lys content and excreted less of it, thereby increasing its digestibility.

The present study also showed that the dietary CP content interacted with BCAA to influence abdominal fat pads. An increase in BCAA % resulted in a reduction of fat pads in RP-diets, whereas elevated BCAA levels in HCP-diets showed the opposite effect. This finding partly agrees with the findings of Ospina-Rojas et al. (2017), where an increase in Leu-to-Lys ratio from 98 to 171 and Val-to-Lys ratio from 53 to 107 decreased fat deposition in broilers fed RP-diets (16% CP) from d 21 to 42. Erwan (2018) also noted a decrease in abdominal fat by increasing Leu from 0% to 0.67% in 18% CP diets. However, at 0.75% Leu, the fat content increased again, indicating a quadratic relationship between BCAA and abdominal fat, similar to the results from the present study. In the current study, quadratic relationships were observed between the fat pad and both NE and BCAA. The data suggests that an increase in BCAA initially raises the fat pad content but then decreases it, regardless of the NE levels. Similarly, a reduction in NE leads to a decrease in the fat pad, regardless of BCAA levels. The finding that reducing energy decreases fat pad is consistent with literature indicating that an increase in feed energy leads to elevated fat deposition, as excess energy consumed is stored as body lipid (Dozier et al., 2006; Maharjan et al., 2021; Sharma et al., 2018).

### 4.3 Correlations between experimental variables

Moreover, correlations between the measured dietary BCAA concentrations and the measurements confirmed that supplementing RP-diets with BCAA higher than standard did not confer advantages to broilers. Consistently, there were negative correlations between BCAA and the performance variables (WG, FI, MEi, and NEi), and a positive correlation between BCAA and FCR. The likelihood of this outcome may be attributed, as discussed earlier, to the depressed energy utilization caused by the excessive inclusion of BCAA, or at least in part, to BCAA antagonisms (Greenhalgh et al., 2022). In the present study, elevating dietary BCAA inclusions was achieved by adding non-bound BCAA—the form that has been shown to be digested fasted than their protein-bound counterparts (Kidd et al., 2021b; Wu, 2009). Therefore, BCAA antagonisms were mostly likely, especially in the RP-diets, based on possible differences in intestinal uptake rates between bound and non-bound BCAA (Greenhalgh et al., 2022).

Furthermore, it is interesting to note that, unlike dietary AME, feed NE correlated with WG, FCR, MEi, NEi, and dietary BCAA, showing no correlation with FI or fat pad. This suggests that the NE system may predict bird performance more accurately than the ME system, as previously mentioned (Musigwa et al., 2021a; Ning et al., 2013; Pesti and Choct, 2023; Wu et al., 2015).

## 5 Conclusion

In conclusion, elevating BCAA levels above current industry standards did not prove beneficial in RP-diets. The sole observed advantage in this study was a reduction in fat pad content. However, this attribute might have come at the cost of the detrimental impact of excess BCAA on feed consumption, and consequently, its adverse effects on energy utilization. On the other hand, reducing energy densities in RP-diets shows promise in improving FI. Nonetheless, the nitrogen pool necessary for synthesizing the protein required for maximum growth might have remained insufficient. The current study also demonstrated the superiority of the NE system to accurately reflect the true energy level available for production. Further research is necessary to fine-tune dietary energy density and nitrogen pool required in RP-diets for optimal performance.

## Credit Author Statement

**Sosthene Musigwa:** Writing – original draft, Methodology, Investigation, Formal analysis, Data curation, Conceptualization. **Pierre Cozannet:** Writing – review & editing, Investigation, Conceptualization. **Mingan Choct:** Writing – review & editing, Resources, Conceptualization. **Shu-Biao Wu:** Writing – review & editing, Project administration, Methodology, Investigation, Formal analysis, Data curation, Conceptualization.

## Declaration of competing interest

We declare that we have no financial and personal relationships with other people or organizations that can inappropriately influence our work, and there is no professional or other personal interest of any nature or kind in any product, service and/or company that could be construed as influencing the content of this paper.
